# Standardized Respiratory Weaning Protocol to Improve Bronchiolitis Length of Stay and Costs

**DOI:** 10.7759/cureus.114783

**Published:** 2026-08-19

**Authors:** Benjamin Traisman, Christina Chen, Stephanie Lam, Paul Severin, Valerie Kalinowski, Sara Murphy, Anne Geistkemper, Kathleen Piotrowski-Walters

**Affiliations:** 1 Hospital Medicine, Lurie Children's Hospital, Chicago, USA; 2 Hospital Pediatrics, Rush University Medical Center, Chicago, USA; 3 Pediatric Critical Care, Rush University Medical Center, Chicago, USA; 4 Respiratory Therapy, Rush University Medical Center, Chicago, USA; 5 PICU Clinical Nursing, Rush University Medical Center, Chicago, USA

**Keywords:** acute bronchiolitis, average length of stay, high flow nasal cannula (hfnc), hospital cost, protocol, quality improvement and patient safety, respiratory weaning, tretment protocol

## Abstract

Background and objectives

High-flow nasal cannula (HFNC) is widely used in the management of bronchiolitis. However, variation in weaning practices may contribute to prolonged hospitalization. At our institution, the average length of stay (LOS) for bronchiolitis admissions was 3.54 days, exceeding the national average. We hypothesized that implementation of a standardized weaning protocol would reduce LOS and associated hospital costs. Our primary aim was to decrease hospital LOS by 20% among patients <24 months of age admitted with bronchiolitis over the course of one year. A secondary objective was to reduce mean total hospitalization costs over this same time period.

Methods

A multidisciplinary task force of physicians, nurses, and respiratory therapists applied Quality Improvement (QI) methodology to identify drivers of prolonged LOS. A standardized respiratory weaning protocol was developed and implemented. Process and outcome data were extracted from the electronic health record and analyzed using run charts. Inclusion criteria were patients admitted for primary diagnosis of bronchiolitis, <24 months of age, and treated with HFNC.

Results

Following implementation and optimization of protocol utilization, the average LOS decreased by 47%, and mean hospitalization cost decreased by 25.5%. Implementation of a standardized, multidisciplinary HFNC weaning protocol was associated with reduced LOS and hospitalization costs.

Conclusions

These findings support protocolized respiratory care as a key driver of high-value care in bronchiolitis management.

## Introduction

Viral bronchiolitis is a leading cause of hospitalization among infants in the United States, accounting for roughly 18% of all infant admissions and placing a massive annual financial burden on the healthcare system that exceeds $700 million nationally [1]. Over the past decade, high-flow nasal cannula (HFNC) has transitioned from an intensive care unit modality to an intervention on general pediatric wards for managing associated moderate-to-severe respiratory distress [2].

Despite its widespread adoption and clinical utility in relieving the work of breathing, large-scale clinical trials have revealed that early or routine initiation of HFNC does not inherently decrease overall intubation rates or shorten a patient's hospital course. Instead, the proliferation of HFNC has been associated with increasing healthcare costs and prolonged hospitalizations [3].

A major driver of this prolonged length of stay (LOS) is the clinical variability that exists surrounding HFNC weaning practices. Weaning decisions are frequently dictated by subjective, individual provider preferences rather than standardized, objective clinical criteria. This lack of structure often leads to delayed weaning, where clinically stable infants remain on escalated respiratory support longer than medically necessary, leading to extended inpatient stays [4]. Recently published national averages of length of stay in hospitalized children with bronchiolitis are between 2.5 and 3.3 days [5]. At our institution, the baseline LOS for bronchiolitis admissions requiring HFNC was 3.54 days, which underscored a clear opportunity to optimize clinical care efficiency.

Utilizing the Quality Improvement (QI) methodology, we convened a multidisciplinary task force comprising pediatric hospitalists, critical care pediatricians, respiratory therapists, and bedside nurses to design and implement a standardized, objective HFNC weaning protocol. Our primary aim was to achieve a 20% reduction in hospital LOS for patients less than 24 months of age admitted with a primary diagnosis of bronchiolitis. As a secondary aim, we sought to evaluate the impact of these standardized clinical pathways on direct total hospitalization costs.

## Materials and methods

**Figure 1:**
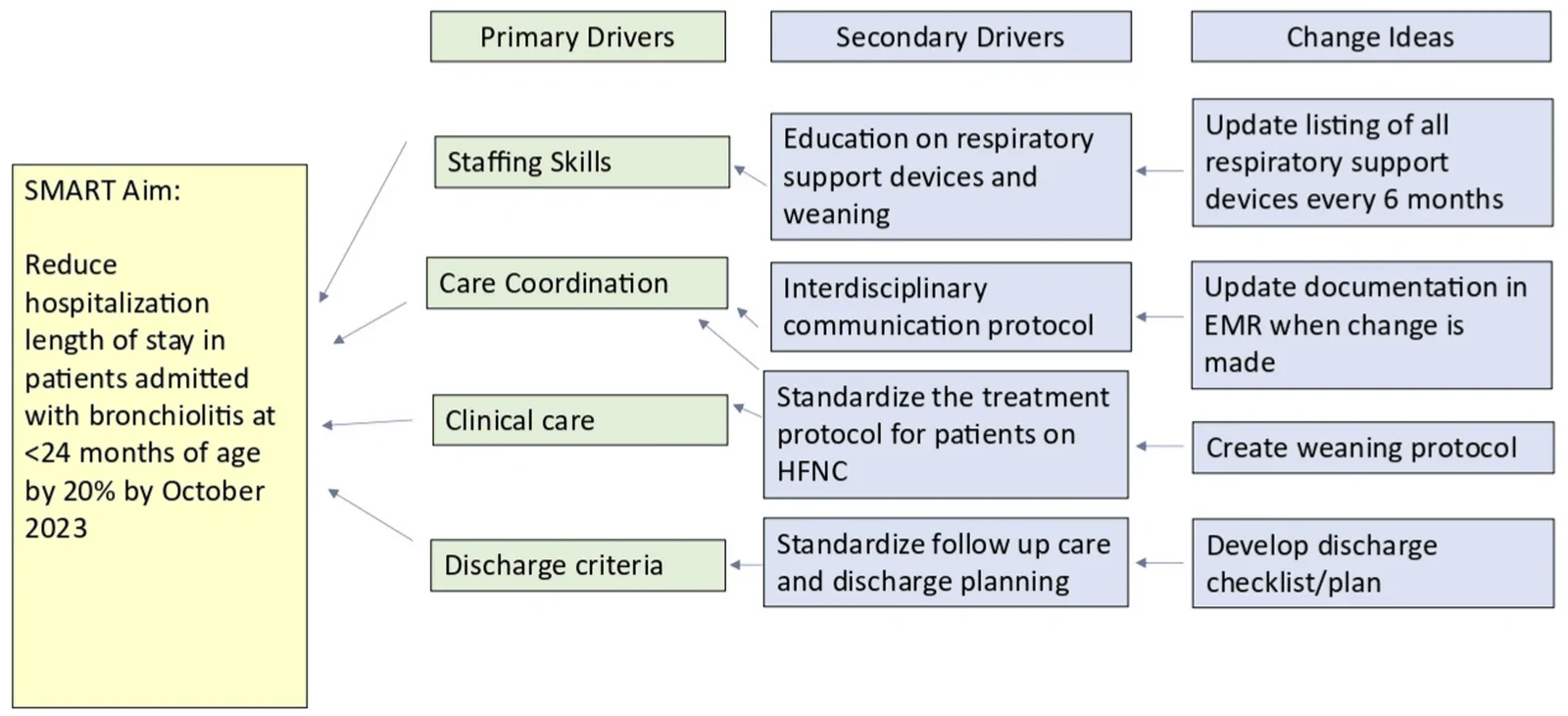
Key driver diagram identifying barriers and change solutions, created by multidisciplinary task force EMR: Electronic Medical Record; SMART: Specific, Measurable, Achievable, Relevant, Time-bound; HFNC: high-flow nasal cannula

**Figure 2:**
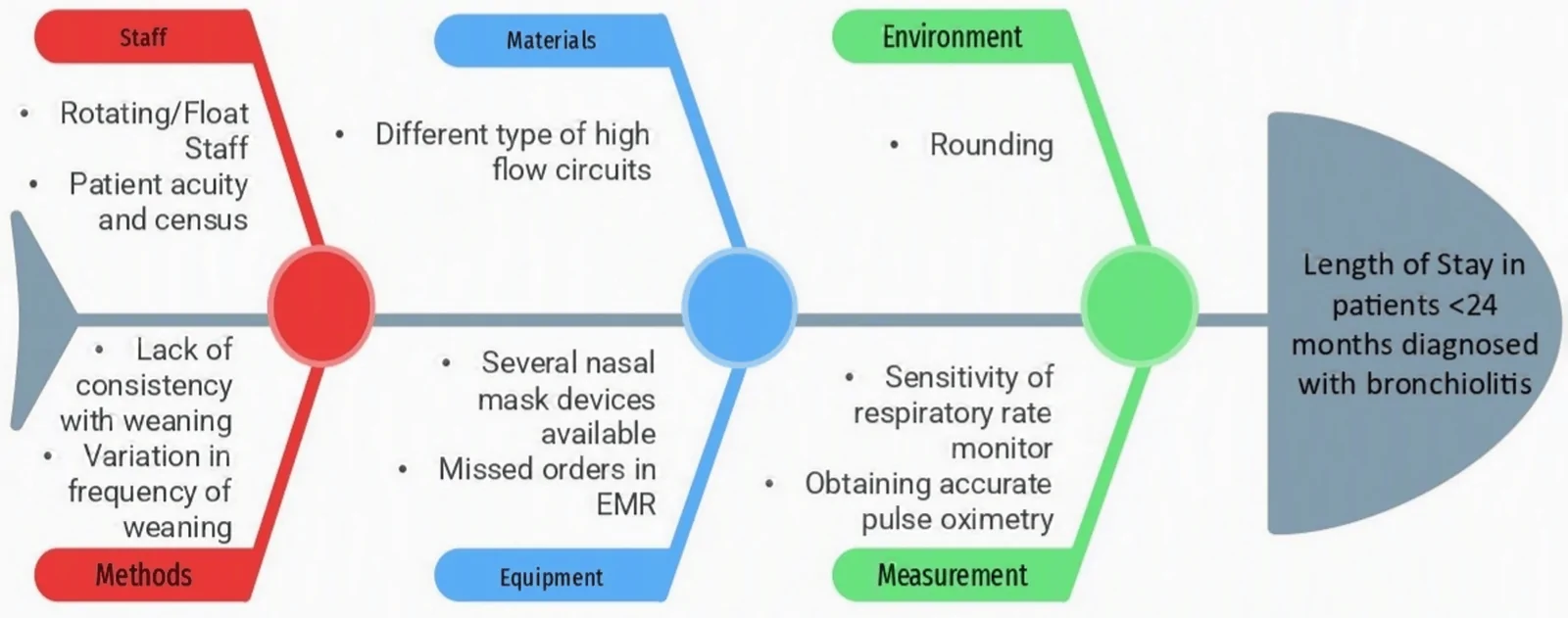
Cause-and-effect diagram: fishbone diagram depicting barriers, created by multidisciplinary task force EMR

Exemption from the Rush University Medical Center Institutional Review Board was obtained before initiating chart review. We conducted a retrospective chart review at an urban, tertiary care pediatric academic medical center. Patients were eligible for inclusion if they were <24 months of age, admitted with a primary diagnosis of bronchiolitis, and required respiratory support via HFNC at any point during the admission. Patients with cyanotic congenital heart disease, chronic lung disease requiring home oxygen, or neuromuscular disorders were excluded. Baseline data for LOS were collected from September 2021 through September 2022 (n=66). Baseline cost data were collected from January 2022 to September 2022 (n=32). Demographic data were not collected. A multidisciplinary task force consisting of pediatric intensivists, hospitalists, nurses, and respiratory therapists identified variability in HFNC weaning as a key driver of prolonged LOS (Fig. 1). A key driver diagram was developed (Fig. 2), and a standardized HFNC weaning protocol was introduced as the primary intervention. As the study progressed, additional Plan-Do-Study-Act (PDSA) cycles were added (Table 1). Data collection was performed for LOS and cost between cycles. The intervention was executed through three PDSA cycles:

**Table 1:** Plan-Do-Study-Act (PDSA) Cycles EHR: Electronic Health Record; LOS: length of stay

Improvement Cycle	Intervention	Time period	Goal
PDSA 1	Creation of standardized weaning protocol	October 2022-August 2023	Decrease LOS by 20% for patients <24 months of age admitted with bronchiolitis
PDSA 2	Targeted multidisciplinary education	August 2024-January 2025	Decrease LOS by 20% from initial baseline for patients <24 months of age admitted with bronchiolitis
PDSA 3	EHR “logic button”	January 2025-May 2026	Decrease LOS by 20% from initial baseline of patients <24 months of age admitted with bronchiolitis

PDSA Cycle 1 (October 2022-August 2023) (n=61): Initial implementation of a standardized HFNC weaning protocol with defined clinical parameters for oxygen titration.

PDSA Cycle 2 (August 2023-January 2025) (n=122): Targeted multidisciplinary education sessions for resident physicians, nurses, and respiratory therapy to improve protocol adherence and improve protocol familiarity.

PDSA Cycle 3 (January 2025-May 2025) (n=22): Integration of a decision-support "logic button" into the Electronic Health Record (EHR) to prompt ordering providers to indicate protocol use during HFNC order entry.

Data collection and key measures

Data were extracted from the EHR for all eligible encounters. Our primary outcome measure was hospital LOS, defined as total days from admission to discharge. The secondary outcome measure was direct hospitalization cost per encounter. LOS and cost data were assessed through chart review following each PDSA cycle to determine if there was a difference in LOS. 

Statistical analysis

A generalized linear mixed-effects model with a Poisson link function was used to analyze the data. The model included a random intercept for each patient to account for within-patient correlation and a fixed effect for the period to assess the impact of the weaning protocol. A generalized linear mixed-effect model was appropriate in this circumstance. There were nine total repeat patients identified throughout the investigation, six in cycle 2 and three in cycle 3. However, each encounter for these patients was deemed its own event and used to evaluate the effectiveness of our intervention. Estimated marginal means and regression coefficients with 95% confidence intervals were calculated to compare differences in LOS across the time periods. This was done for both length of stay and hospitalization cost data. Statistical significance was defined as p < 0.05. 

Run charts were used to assess sustained changes in LOS across PDSA cycles. The median LOS served as the centerline. Shifts and trends were evaluated using standard run chart rules.

## Results

**Figure 3:**
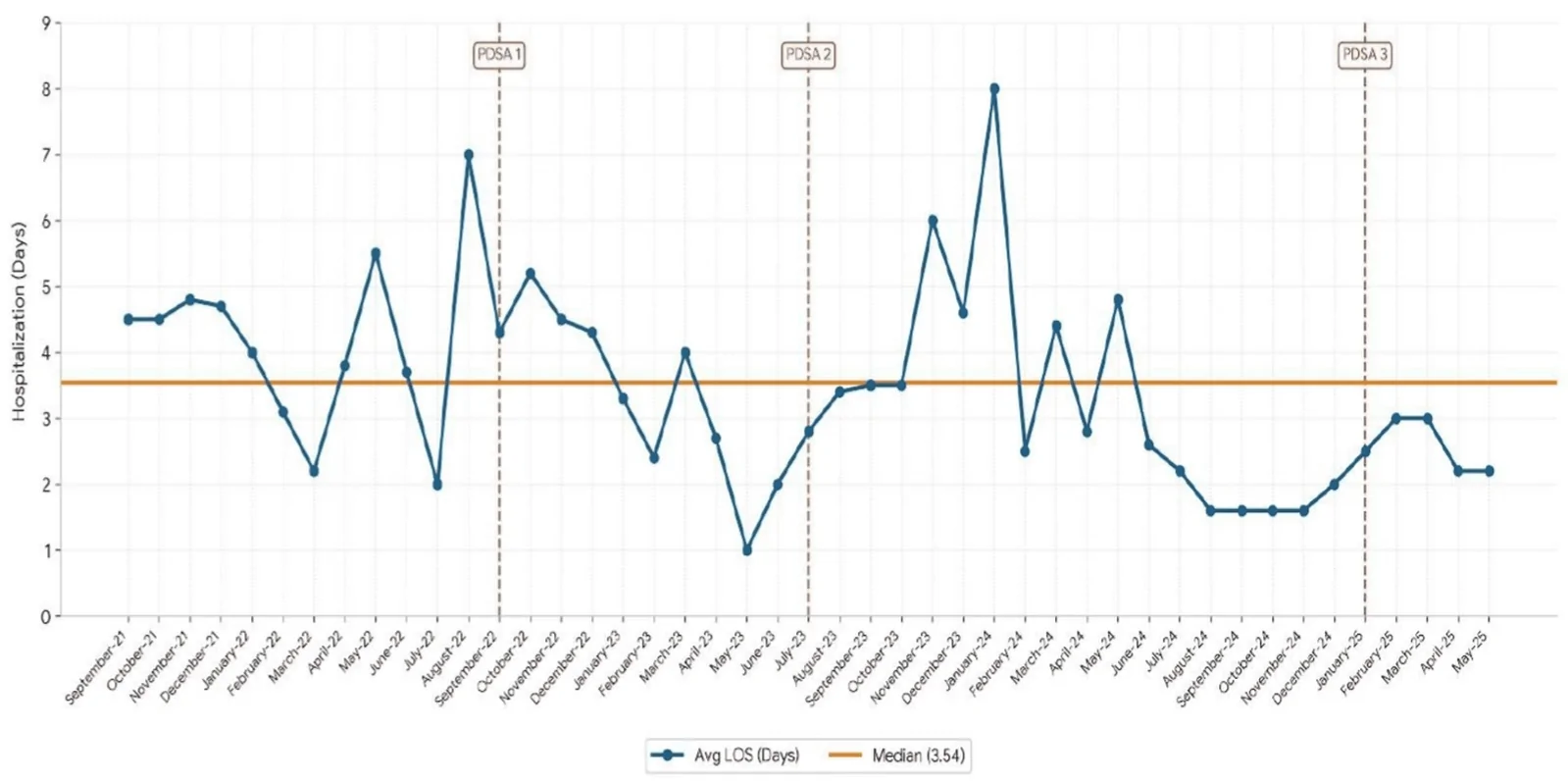
Run-chart with length of hospital stay in days from baseline through PDSA cycles 1, 2, and 3. LOS: length of stay; PDSA: Plan-Do-Study-Act

For pre-intervention (baseline) LOS data, 66 patients (n=66) met inclusion criteria, and for pre-intervention (baseline) cost data, 32 patients (n=32) met inclusion criteria. For LOS, 212 patients (n=212) met inclusion criteria over the full post-intervention phase. For cost data, 212 patients (n=212) met inclusion criteria over the full post-intervention phase. Post-intervention patients were analyzed sequentially across PDSA cycles: PDSA 1 (n=61), PDSA 2 (n=122), PDSA 3 (n=24). The baseline mean LOS was 3.54 days, as determined after the pre-intervention chart review. Implementation of PDSA cycle 1 (protocol delivery) and PDSA cycle 2 (multidisciplinary education) resulted in modest reductions in LOS of 6.6% (rate ratio 0.934, p=0.660, 95% CI 0.691-1.264, Wald z-score -0.44) and 0.5% (rate ratio 0.995, p=0.973, CI 0.768-1.290, Wald z-score -0.04), respectively. Neither intervention produced a sustained shift below the median when plotted on a run chart (Fig. 3).

Following PDSA cycle 3 (EHR integration), LOS decreased by 47%, which was statistically significant (rate ratio 0.510, p= 0.003, 95% CI 0.325-0.800, Wald z-score -2.93). This sustained decrease is evident in the run chart after the PDSA 3 time mark (Fig. 3). By the end of the study period, the mean LOS was 1.89 days.

Baseline mean hospitalization costs were $8,093. After implementation of the standardized weaning protocol (PDSA cycle 1), mean costs decreased to $7,063, representing a 12.7% reduction; however, this change did not reach statistical significance (rate ratio 0.873, p =0.525, 95% CI 0.574-1.328, Wald z-score -0.63). Following the multidisciplinary education intervention (PDSA cycle 2), mean costs rose to $8,257, exceeding the baseline mean.

The largest reduction in cost occurred after implementation of the EHR logic button (PDSA cycle 3), where mean hospitalization costs reached a study low of $6,025. While this represented a 25.5% reduction from baseline and a 27% reduction from the PDSA 2 peak, due to the substantial variability in cost data, this reduction did not reach statistical significance (rate ratio 0.730, p=0.125, 95% CI 0.488-1.091, Wald z-score -1.53). 

For balancing measures, we evaluated 30-day readmission in the baseline period and over the course of each PDSA cycle. In the baseline period, there were five readmissions with a diagnosis of bronchiolitis. There were five readmissions following PDSA 1, seven readmissions following PDSA 2, and no readmissions following PDSA 3.

## Discussion

This quality improvement initiative demonstrated that implementation of a standardized HFNC weaning protocol, particularly when integrated into the Electronic Health Record, was associated with substantial reductions in length of stay for patients with bronchiolitis. While protocol development and educational interventions help reduce variability in clinical practice, integration of protocols directly into the EHR may be a more effective strategy for sustaining changes in workflow to improve reliability and adherence.

This is highly consistent with the hierarchy of controls in patient safety, which discusses the inherently more effective nature of system-level forcing functions, automation, and engineered barriers versus reliance on human memory, vigilance, or passive compliance [6].

The limited impact during our first two Plan-Do-Study-Act (PDSA) cycles highlights a well-documented challenge in healthcare quality improvement: the difficulty of sustaining behavior changes through passive interventions such as education alone. Passive dissemination of evidence-based clinical practice guidelines has been repeatedly shown to produce only minimal or transient changes in the uptake of new practices [7]. The modest 6.6% reduction in LOS following PDSA cycle 1 likely reflects the robust nature of multidisciplinary involvement in protocol development, which succeeded in expanding our base of key stakeholders and generating initial clinical buy-in [8,9].

However, the much smaller improvement observed during PDSA cycle 2 (0.5%) indicates challenges in maintaining consistent educational reinforcement amid shifting clinical staff, rotating resident classes, and competing inpatient priorities. Without an active forcing function embedded in the daily workflow, practice patterns frequently revert toward baseline variation over time.

**Figure 4:**
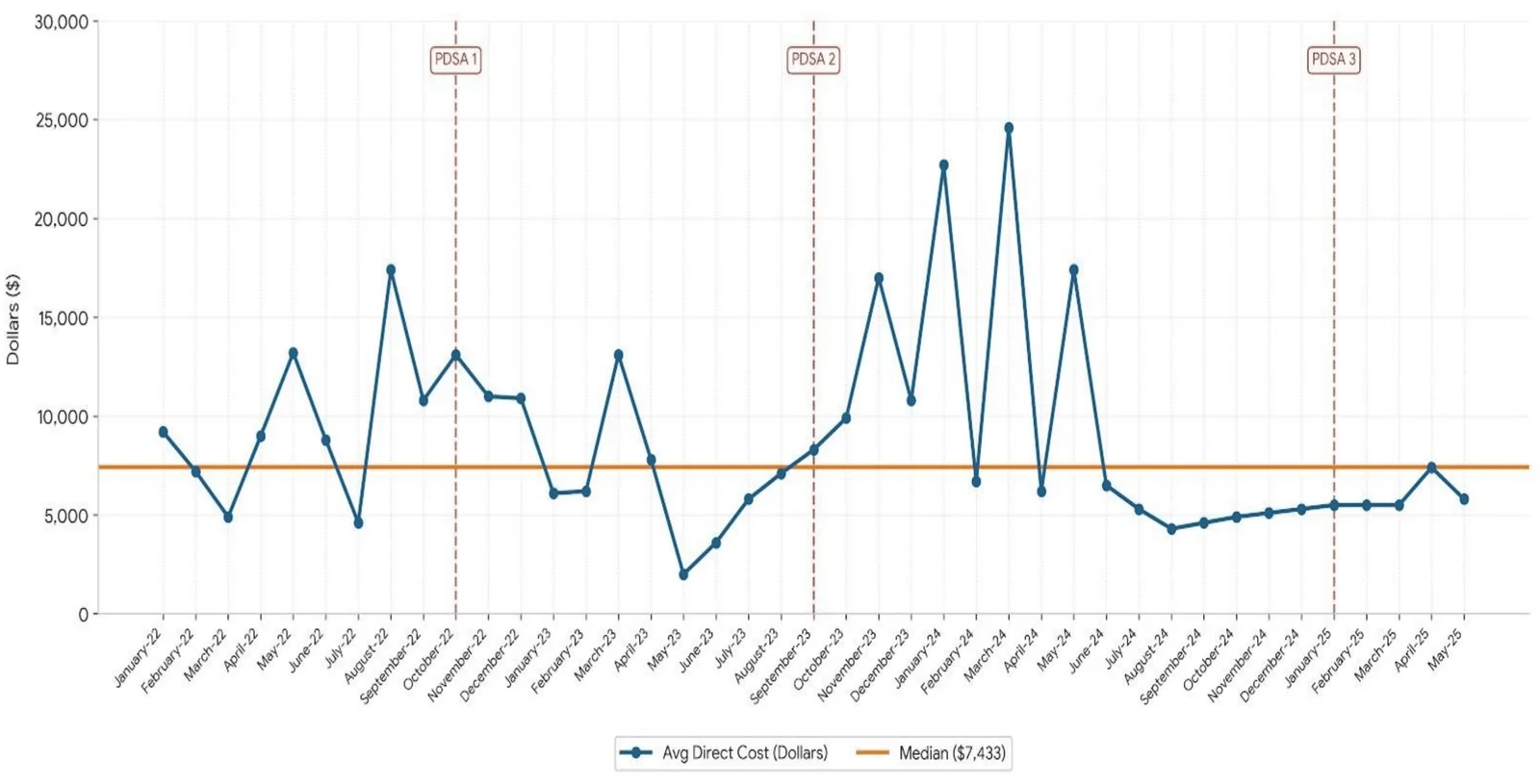
Run chart of hospital direct costs from baseline through PDSA cycles 1, 2, and 3 PDSA: Plan-Do-Study-Act

The true inflection point of our study occurred with automated EHR integration, after which hospitalization costs followed a similar downward trajectory to LOS. Although strict statistical significance was not achieved for every secondary metric, the absolute magnitude of cost reduction suggests a clinically and operationally meaningful improvement in resource allocation. Cost improvement is further evident when viewed on a run chart with sustained shift starting through PDSA 3 (Fig 4). Our success with electronic integration aligns with previous pediatric QI literature demonstrating that standardized, protocol-driven HFNC initiation and automated weaning order sets significantly reduce overall hours of respiratory support and curb the overutilization of redundant respiratory therapies without increasing adverse bounce-back rates [10,11]. Protocol adherence was 22/24 patients admitted during PDSA 3. While robust protocol usage was encouraging, this may have been due to the Hawthorne effect.

Several limitations should be considered when interpreting these findings. First, the innate seasonal variation of viral bronchiolitis can severely confound longitudinal LOS trends. While seasonal fluctuations were evident in our run chart, it is notable that the steep improvement observed during PDSA cycle 3 began precisely during the peak of the typical viral season and was sustained throughout the remainder of the study period, suggesting the protocol's efficacy. There was an extended gap between PDSA cycle 2 and cycle 3, as implementing a device into the EHR required systems approval that delayed the intervention. However, as mentioned previously, the intervention was launched during peak viral season, which dampens the further impact of seasonal variability. Second, other clinical factors beyond primary bronchiolitis may have influenced LOS and hospitalization costs; these include secondary bacterial infections, severe gastrointestinal symptoms, or concomitant chronic conditions that were not excluded from our sample. Large-scale database studies have heavily documented that infants with underlying comorbidities experience vastly higher healthcare service utilization, longer hospital stays, and more frequent ICU admissions than otherwise healthy infants [12]. These comorbidities naturally deviate from standard weaning pathways and can skew financial metrics. Our absence of demographic data also limits our ability to analyze how demographics play a role in weaning from respiratory support. Finally, as a single-center study, our overall sample size and variation in sample sizes between interventions may have limited our statistical power to detect statistically significant differences in complex cost outcomes [13,14].

## Conclusions

Despite these limitations, this initiative demonstrated that implementation of a standardized HFNC weaning protocol, particularly when integrated into the EHR, was associated with significant reductions in hospital LOS and meaningful decreases in hospitalization costs without increasing readmissions. These findings suggest that combining protocolized care with workflow-integrated decision support may be an effective strategy for improving value in inpatient bronchiolitis management. Further exploration can also be done to simplify the protocol to improve adherence, incorporate weight-based flow rates, and add HFNC Holiday. Future multi-centered studies are needed to validate these results and assess broader applicability.
